# The pattern of *kdr* mutations correlated with the temperature in field populations of *Aedes albopictus* in China

**DOI:** 10.1186/s13071-021-04906-z

**Published:** 2021-08-16

**Authors:** Hanming Chen, Qiuming Zhou, Haowei Dong, Hao Yuan, Jie Bai, Jingpeng Gao, Feng Tao, Hui Ma, Xiangyu Li, Heng Peng, Yajun Ma

**Affiliations:** 1 College of Naval Medicine, Naval Medical University, Shanghai, China; 2 Department of Medical Microbiology and Parasitology, College of Basic Medical Sciences, Naval Medical University, Shanghai, China; 3 Sixth Medical Center of Chinese PLA General Hospital, Beijing, China

**Keywords:** *Aedes albopictus*, Voltage-gated sodium channel gene, *Kdr* mutations, Distribution pattern, Pyrethroid resistance

## Abstract

**Background:**

*Aedes albopictus* is the primary vector of dengue fever in China. This mosquito species has a wide distribution range in China and can be found in the tropical climate zones of southern provinces through to temperate climate zones of northern provinces. Insecticides are an important control method, especially during outbreaks of dengue fever, but increasing insecticide resistance raises the risk of failure to control vector-borne diseases. Knockdown resistance (*kdr*) caused by point mutations in the voltage-gated sodium channel (*VGSC*) gene is a key mechanism that confers resistance to pyrethroids. In this study we explored the characteristics and possible evolutionary trend of *kdr* mutation in *Ae. albopictus* based on analysis of the *kdr* mutations in field populations of mosquitoes in China.

**Methods:**

A total of 1549 adult *Ae. albopictus* were collected from 18 sites in China from 2017 to 2019 and 50 individuals from three sites in the 1990s. A fragment of approximately 350 bp from part of the S6 segment in the *VGSC* gene domain III was amplified and sequenced. Using TCS software version 1.21A, we constructed haplotypes of the *VGSC* gene network and calculated outgroup probability of the haplotypes. Data of annual average temperatures (AAT) of the collection sites were acquired from the national database. The correlation between AAT of the collection site and the *kdr* mutation rate was analyzed by Pearson correlation using SPSS software version 21.0.

**Results:**

The overall frequency of mutant allele F1534 was 45.6%. Nine mutant alleles were detected at codon 1534 in 15 field populations, namely TCC/TCG (S) (38.9%), TTG/CTG/CTC/TTA (L) (3.7%), TGC (C) (2.9%), CGC (R) (0.3%) and TGG (W) (0.1%). Only one mutant allele, ACC (T), was found at codon 1532, with a frequency of 6.4% in ten field populations. Moreover, multiple mutations at alleles I1532 and F1534 in a sample appeared in five populations. The 1534 mutation rate was significantly positively related to AAT (Pearson correlation: *r*_(18)_ = 0.624, *P* = 0.0056), while the 1532 mutation rate was significantly negatively related to AAT (Pearson correlation: *r*_(18)_ =  − 0.645, *P* = 0.0038). Thirteen haplotypes were inferred, in which six mutant haplotypes were formed by one step, and one additional mutation formed the other six haplotypes. In the samples from the 1990s, no mutant allele was detected at codon 1532 of the *VGSC* gene. However, F1534S/TCC was found in HNHK94 with an unexpected frequency of 100%.

**Conclusions:**

*Kdr* mutations are widespread in the field populations of *Ae. albopictus* in China. Two novel mutant alleles, F1534W/TGG and F1534R/CGC, were detected in this study. The 1534 *kdr* mutation appeared in the population of *Ae. albopictus* no later than the 1990s. The F1534 mutation rate was positively correlated with AAT, while the I1532 mutation rate was negatively correlated with AAT. These results indicate that iInsecticide usage should be carefully managed to slow down the spread of highly resistant *Ae. albopictus* populations, especially in the areas with higher AAT.

**Graphical abstract:**

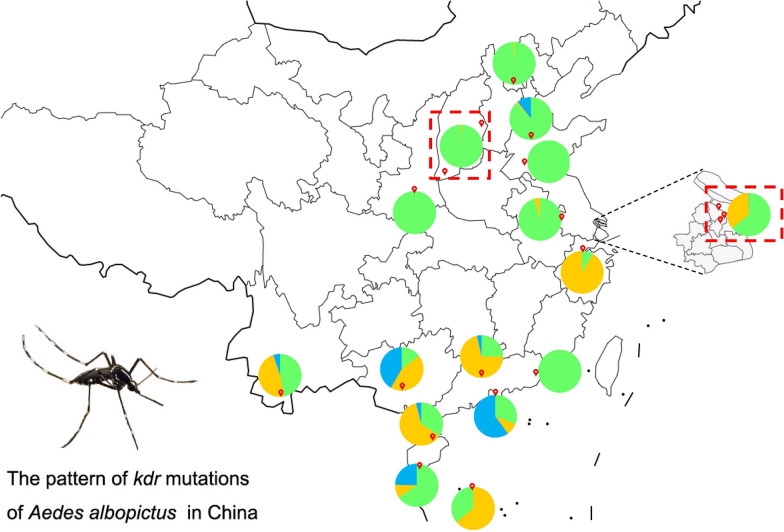

**Supplementary Information:**

The online version contains supplementary material available at 10.1186/s13071-021-04906-z.

## Background

Dengue fever is one of the most widespread mosquito-borne diseases worldwide, with an estimated 390 million dengue virus infections occurring annually worldwide [1]. In China, dengue fever has become an important public health emergency, with 22,599 dengue fever cases reported in 2019 and the distribution area reaching an unprecedented level [2, 3]. Due to the scarcity of therapeutic drugs and an effective vaccine, vector control is still the main measure to prevent this disease [4].

*Aedes aegypti* and *Ae. albopictus* are the main vectors of dengue fever [5]. Although *Ae. aegypti* is more susceptible to the dengue virus, *Ae. albopictus* is considered to be the main vector of dengue fever in China due to its wide distribution range [6]. *Aedes albopictus* is distributed from Liaoning Province in the north to Hainan Province in the south of China, covering tropical, subtropical and temperate climate zones [6]. An effective insecticide is an essential method to control the *Ae. albopictus* vector, especially during outbreaks of mosquito-borne diseases.

With the advantages of high efficiency and low toxicity, pyrethroid insecticides have been widely used to control mosquitoes in China since the 1980s [7]. In the context of an emphasis on integrated control in the general approach to *Ae. albopictus* control, pyrethroid insecticides were sprayed heavily during past outbreaks of dengue fever to quickly control the epidemic [8, 9]. However, the long-term and mass application of insecticides has led to the serious problem of insecticide resistance. High-level and multiple resistance to pyrethroid insecticides has been observed in many *Ae. albopictus* field populations in China [10–12].

The target of pyrethroids is the voltage-gated sodium channel gene (*VGSC*), which interferes with electrical signaling in the nervous system, leading to paralysis, an effect known as knockdown [13]. Target insensitivity is a critical mechanism of pyrethroid resistance development, caused by mutations in *VGSC*, the so-called knockdown resistance mutation (*kdr* mutation) [14, 15].

The *kdr* mutation in *Ae. albopictus* was first detected in Singapore in 2009, from samples collected in the field by Kasai et al. [16]. Thereafter, different *kdr* mutations were reported in various field-collected *Ae. albopictus* populations [17], such as V1016G in Italy and Vietnam [18]; F1534C in Singapore, Brazil, India, Italy, Vietnam and Greece [16, 19, 20]; F1534L in the USA [19]; F1534S in the USA, Italy and Vietnam [18, 19]; and I1532T in Italy [19]. In China, our group was the first to report the presence of the F1534S and F1534L mutant alleles, identified in *Ae. albopictus* collected from Haikou, Hainan [21, 22]. This was followed by reports of the F1534S/L/C, and I1532T mutations in *Ae. albopictus* populations collected in Shanghai, Jiangsu, Yunnan, Beijing, Shandong and Guangdong [19, 23–30]. The V1016G and D1736Y mutations were also detected in certain populations [27, 28].

Several *kdr* mutations have been identified, some of which have been confirmed to confer insecticide resistance [12, 31, 32]. However, how these mutations appear, accumulate and disperse in field populations remains a puzzling issue that is attracting the attention of researchers. Moreover, the evolutionary trend of the *kdr* mutation may be the key to determining the strategy of insecticide application in the future.

In this study, we studied 18 field populations collected from sited ranging in location from the north to south of China and three populations collected in the 1990s. The types and frequencies of *kdr* mutations were surveyed and analyzed to explore the characteristics and possible evolutionary trend of *kdr* mutations.

## Methods

### Sample collection and species identification

**Fig. 1:**
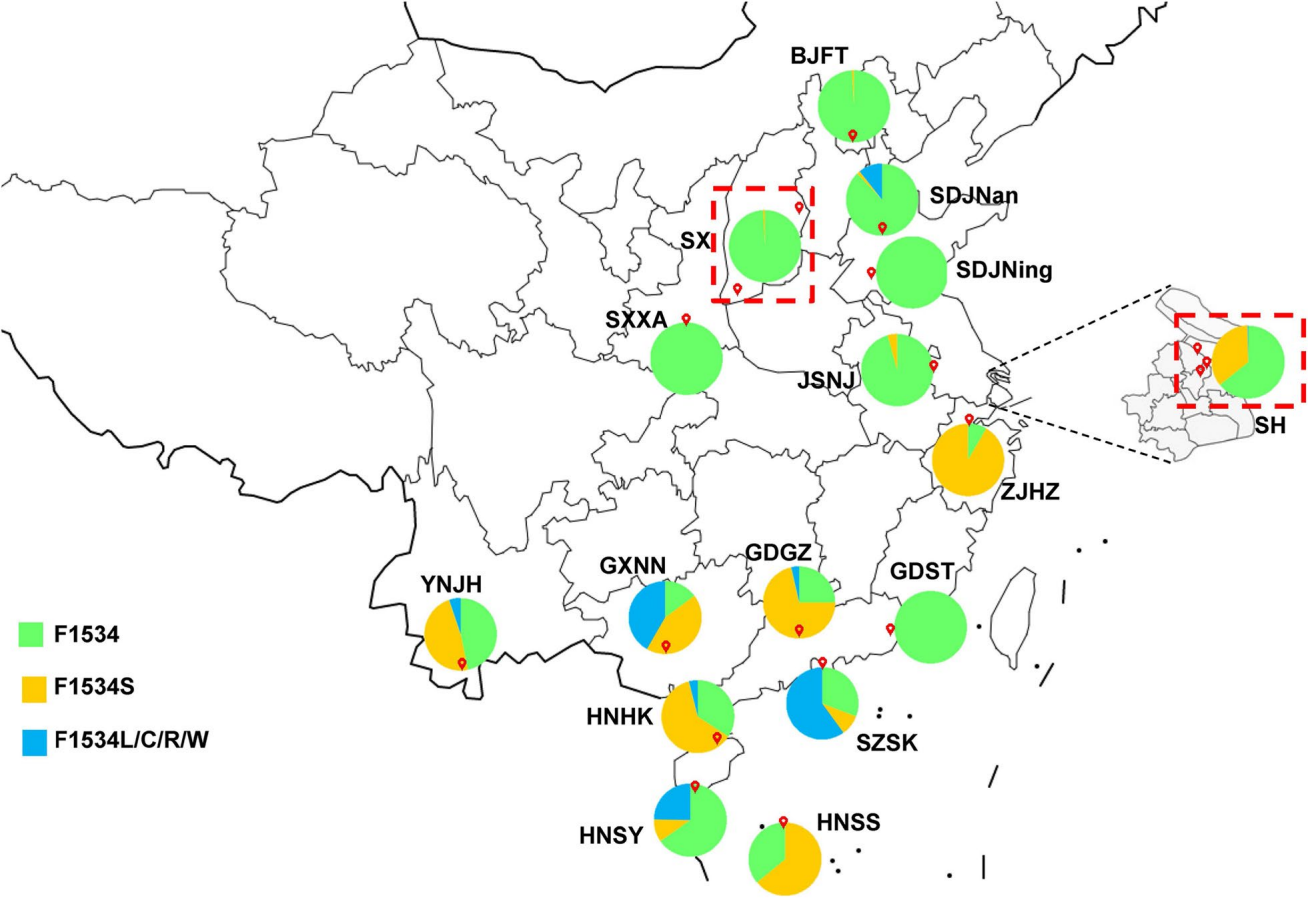
Schematic map of collection sites for *Aedes albopictus* in China and the codon composition of the mutant 1534 alleles of the voltage-gated sodium channel gene (*VGSC*) gene in the samples. Red balloons indicate the sampling sites (for details see Additional file 1: Table S1). The codon composition of the 1534 allele in each site is represented by a pie chart, with green indicating the wild-type F1534 allele; yellow, mutant allele F1534S; and blue, mutant allele F1534L/C/R/W (for details see Additional file 1: Table S2). The red-dashed squares indicate multiple mosquito collection points in a group

Eighteen field populations of *Ae. albopictus* were collected from 13 provinces (municipalities) in China from 2017 to 2019 (Fig. 1). Larvae and pupae were scooped from more than five breeding sites (in a collection site), brought back to the laboratory and reared to adults under standard conditions (26 ℃ ± 1 °C, 65% ± 5% relative humidity, photoperiod of 12/12 h [light/dark]). Adult mosquitoes were collected using aspirators, BG-traps (Biogents AG, Regensburg, Germany) or light traps (Houji Dianzi, China) in outdoor environments near humans.

To explore the historical changes in *kdr* mutations in *Ae. albopictus*, we also used samples from three field populations collected in the 1990s, namely HNHK94 (Haikou, 1994), GDGZ95 (Guangzhou, 1995) and SCCD97 (Chengdu, 1997). These samples were dried and stored at – 20 ℃ in our laboratory until used for *kdr* mutation detection in this study.

Information on collection is summarized in Additional file 1: Table S1. Species of adult *Aedes* mosquitoes were identified on the basis of morphological characteristics [33] and identification was confirmed by molecular markers [34].

### Annual average temperature of collection sites

The China National Meteorological Science Data Center (CNMSDC, http://data.cma.cn) provided information on the annual average temperature (AAT) and average monthly temperature (AMT) in January and July at the collection sites from January 2010 to December 2019 (Additional file 1: Table S2).

### DNA extraction

Genomic DNA was extracted from adult mosquitoes using the DNAzol reagent (Invitrogen, Thermo Fisher Scientific, Waltham, MA, USA) according to the manufacturer’s instructions. Briefly, a single mosquito was put in a 1.5-ml Eppendorf tube and efficiently ground to powder by first being frozen in liquid nitrogen and then crushed with a pestle. For lysis, 100 μl of DNAzol was added to the ground sample, and the supernatant was transferred to another Eppendorf tube. After adding 50 μl ethanol, the mixed solution was stored overnight at − 20 ℃. DNA, following which the solution was adsorbed through a nucleic acid adsorption column. After washing with 70% ethanol, the DNA was eluted from the column with ddH_2_O. The DNA concentration was quantified by measure absorption using a Nano-500 Micro-Spectrophotometer (Allsheng, Hangzhou, China) at OD_280_.

### PCR and *kdr* allele detection

A fragment of approximately 350 bp of the S6 segment in the *VGSC* gene domain III was amplified and sequenced using a PCR kit (Aidlab, Beijing, China) using primers aegSCF7 (5′-AGG TAT CCG AAC GTT GCT GT-3′) and aegSCR8 (5′-TAG CTT TCA GCG GCT TCT TC-3′) [12, 16]. The PCR reaction was conducted in a Veriti 96-well Thermal Cycler (Applied Biosystems, Thermo Fisher Scientific), set at the following cycling conditions: an initial denaturation step at 94 ℃, 2 min; then amplification at 94 ℃/30 s, 52 ℃/30 s, 72 ℃/30 s for 35 cycles; with a final extension step at 72 ℃ for 8 min. After electrophoresis, PCR products were purified from the gel excisions and sequenced from both directions. Sequences were aligned and analyzed using DNASTAR Lasergene v.12.0 software with the partial sequence of sensitive *Ae. albopictus* isolate FS38 *VGSC* IIIS6 (NCBI: MN433857.1) as a reference [35].

### The haplotype network of *VGSC* gene

The haplotypes of the *VGSC* gene were inferred by the alleles in codons 1532 and 1534. The genealogical relationship among haplotypes at the population level was estimated using the method of Templeton et al. [36]. A haplotype network was constructed, and an outgroup probability of the haplotype was calculated based on statistical parsimony using TCS version 1.21 software [37].

### Statistical analysis

The allele frequency of *kdr* mutations was calculated as follows:

$$\mathrm{R}=\frac{k}{n\times 2}\times 100\%,$$

where R is the allele frequency,* k* represents the number of alleles and* n* represents the sample size.

The mutation frequency was defined as the frequency of wild-type–mutant heterozygotes and mutant–mutant homozygotes, which was calculated as follows:

$$\mathrm{M}=\frac{a+b}{n}\times 100\%,$$

where M is the mutation frequency,* a* represents the number of wild-type–mutant samples,* b* represents the number of mutant–mutant samples and* n* is the sample size.

The correlation between the AAT of the collection site and the *kdr* mutation rate was analyzed by Pearson correlation using SPSS version 21.0 software (SPSS IBM Corp., Armonk, NY, USA). The difference in the mutation rate between groups was calculated by one-way analysis of variance (ANOVA) using SPSS version 21.0.

## Results

### Mosquito samples

A total of 1549 adult *Ae. albopictus* were collected from 18 sites in China from 2017 to 2019 (Additional file 1: Table S3). Information on the *kdr* mutation of five populations (SDJNan, SHBS, SHYP, ZJHZ and HNHK) has been used earlier in previous analyses and published [12, 30]. In addition to these five populations, we used 50 individuals from three sites that had been collected in the 1990s, with the aim to to explore the historic change in the *kdr* mutation; these populations were HNHK94 (Haikou 1994, *n* = 12), GDGZ95 (Guangzhou 1995, *n* = 18) and SCCD97 (Chengdu 1997, *n* = 20).

### F1534 mutation rate at codon

The wild-type codon at the 1534 allele was TTC (F). The overall averaged frequency of a mutant allele F1534 across all collection sites was 45.6%, which is the codon with the highest mutation frequency and highest prevalence in *VGSC* of *Ae. albopictus*. Nine mutant alleles were detected: TCC/TCG (S) (38.9%), TTG/CTG/CTC/TTA (L) (3.7%), TGC (C) (2.7%), CGC (R) (0.3%) and TGG (W) (0.1%). The mutant allele TCC had the highest frequency (38.7%), whereas the mutant allele TGG had the lowest (0.1%) (Fig. 1; Additional file 1: Table S3). Fifteen genotypes were detected (Additional file 1: Table S4), including the wild-type genotype F/F; four wild-type–mutant heterozygotes F/L, F/S, F/C, and F/R; and ten mutant genotypes L/L, C/C, S/S, C/R, L/C, L/R, C/W, S/R, L/S, and S/C. The frequencies of the wild-type genotype, wild-type–mutant heterozygote and mutant genotype were 40.6, 27.6 and 31.8%, respectively (Additional file 1: Table S4), indicating that 59.4% of adult *Ae. albopictus* carried mutations at the 1534 codon of the *VGSC* gene.

### Pattern of F1534 mutation in the field populations

Of the 18 field populations studied, 15 had F1534 mutations (Additional file 1: Table S3). The highest frequency was in the ZJHZ population (91.6%), followed by the population collected at GXNN (85.2%), GDGZ (75.0%), SZSK (69.2%), HNHK (65.8%), HNSS (63.9%) and YNJH (53.3%) (see Fig. 1 for locations) and, finally, by eight populations with frequencies < 50%. Three populations carried no mutation in 1534, namely, GDST, SXXA, and SDJNing (Additional file 1: Table S3).

In terms of mutant allele types, the SZSK population carried the most types of mutant alleles with eight, followed by GXNN, YNJH and HNSY with four, GDGZ and SHYP with three and HNHK and SDJNan with two. In the other seven populations, only one mutant allele, F1534S/TCC, was detected (Additional file 1: Table S3).

1534TCC/S was the most common mutant allele and was detected in all 15 populations. Four mutant alleles, namely CTG/L, CGC/R, TGG/W and TTA/L, were found in only one population; among these, CTG/L (3.3%), CGC/R (6.7%) and TGG/W (2.5%) were found only in SZSK, and TTA/L (10.7%) was identified only in SDJNan.

### I1532 mutation rate at codon

With the exception of the wild-type allele ATC (I), only one mutant allele ACC (T) was found at codon 1532 (Additional file 1: Table S3). Compared with the 1534 codon, the 1532 codon had a much lower mutation frequency of 6.4%. Three genotypes were found, namely wild-type genotype I/I (88.6%), wild-type–mutant heterozygote I/T (10.1%) and mutant genotype T/T (1.4%) (Additional file 1: Table S5).

### F1532 mutation pattern in the field populations

**Fig. 2:**
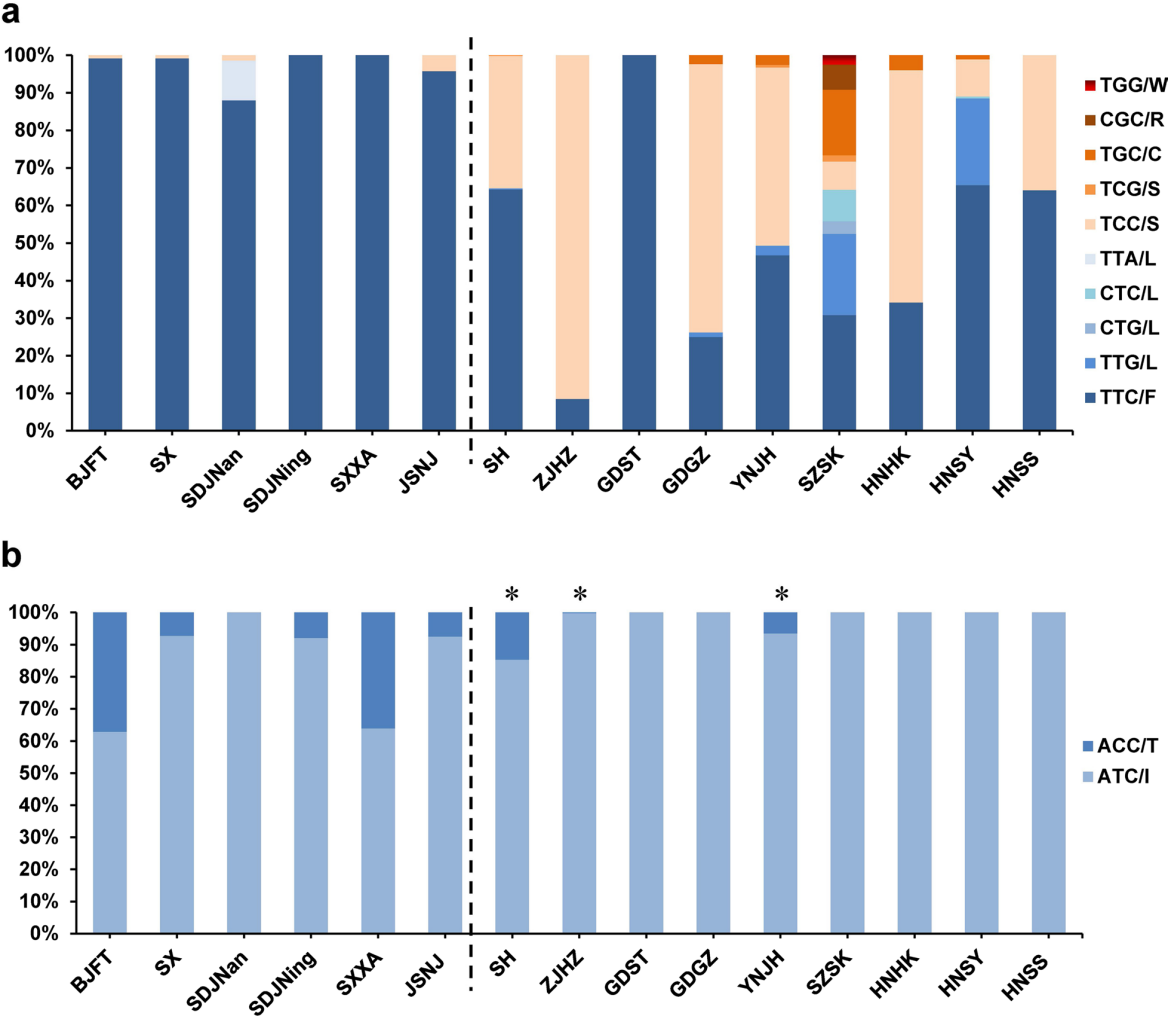
Histogram of allele frequency at codons 1532 (**a**) and 1534 (**b**) of the *VGSC* gene in *Aedes albopictus* populations. SH represents the pool of three populations (SHBS, SHYP and SHGQ). For detailed information, see Additional file 1: Tables S1 and S2. The populations on the left of the dotted line are from collection sites in central and northern China, and the populations on the right side are from sites in southern China. The horizontal axis indicates the populations (see Fig. 1 for details), while the vertical axis denotes the allele frequency. Populations marked with an asterisk indicate the co-occurrence of both mutations (codons 1532 and 1534)

The mutant allele I1532T was detected in ten field populations: YNJH, BJFT, SDJNing, SHBS, SHYP, SHGQ, ZJHZ, SXXA, JSNJ and SX. The highest frequency was 37.1% in the BJFT population, followed by 36.0% in the SXXA population (Additional file 1: Table S3; Fig. 2). The mutant homozygous 1532 T/T was found in six populations, namely BJFT (13.8%), SXXA (12.0%), SHYP (5.1%), SHGQ (2.4%), SDJNing (2.3%) and SHBS (0.7%) (Additional file 1: Table S5).

### Multiple mutations at codon 1532 and 1534

Multiple mutations at I1532 and F1534 appeared in five populations, namely YNJH, SHBS, SHYP, SHGQ and ZJHZ. The highest frequency of co-occurrence of mutations was 21.4% in the SHYP population, followed by 15.1% in the SHGQ population. Three types of combined mutations were present: I/T + F/S, I/T + S/S and T/T + F/L. However, no individuals with mutant homozygotes at both codons were found (Table 1).

**Table 1:** Frequency of co-occurrence of I1532T + F1534S genotypes of the voltage-gated sodium channel gene in *Aedes albopictus* populations collected in China

Loci		Population^a^				
I1532	F1534	SHBS	SHGQ	SHYP	YNJH	ZJHZ
I/T	F/S	5 (3.6%)	19 (15.1%)	19 (19.4%)	6 (7.8%)	0
I/T	S/S	0	0	0	0	1 (0.4%)
T/T	F/L	0	0	2 (2.0%)	0	0

Data are presented as the number with the percentage of co-occurrence of the I1532T + F1534S genotype given in parentheses

^a^See Fig. 1 for details on location of populations

### Geographic distribution of *kdr* mutants of *Ae. albopictus* in China

Interestingly, the pattern of *kdr* mutations in *Ae. albopictus* seems to be related to geographical factors. F1534S/L/C/R/W mutant alleles were mainly found in southern and central China (Fig. 2a), while the I1532T mutant allele was mainly found in populations from northern and central China (Fig. 2a). Were the field populations to be divided into southern and northern groups by the January zero degree isotherm 32°N (Fig. 2), then there would be a significant difference in the 1534 mutation rate (ANOVA: *F*_(1, 16)_ = 12.55, *P* = 0.0027) and the 1532 mutation rate (ANOVA: *F*_(1, 16)_ = 5.67, *P* = 0.0300) between the two groups.

To further explore this phenomenon, we collected data on the AAT and AMT in January and July at the collection sites (Additional file 1: Table S2). The 1534 mutation rate was significantly positively related to AAT (Pearson correlation: *r*_(18)_ = 0.611, *P* = 0.0071) and AMT in January (Pearson correlation: *r*_(18)_ = 0.615, *P* = 0.0066), while the 1532 mutation rate was significantly negatively related to AAT (Pearson correlation: *r*_(18)_ =  − 0.608, *P* = 0.0074) and AMT in January (Pearson correlation: *r*_(18)_ = 0.607, *P* = 0.0075). Neither the 1534 nor 1532 mutation rate was related to AMT in July.

Notably, the ZJHZ population was collected during the 2019 Hangzhou dengue fever epidemic. At that time, many insecticides had been used, so the collected mosquitoes were intended to be a screened resistant population. The collection site of the GDST population is close to the sea; subsequent oral interviews revealed that almost no insecticides were used in the local area. Removal of the ZJHZ and GDST populations from the correlation analysis resulted in the 1534 mutation rate being significantly positively related to AAT (Pearson correlation: *r*_(16)_ = 0.817, *P* = 0.0001) and the 1532 mutation rate being significantly negatively related to AAT (Pearson correlation: *r*_(16)_ =  − 0.614, *P* = 0.0114).

### Genealogical relationships among the haplotypes of the *VGSC* gene

**Fig. 3:**
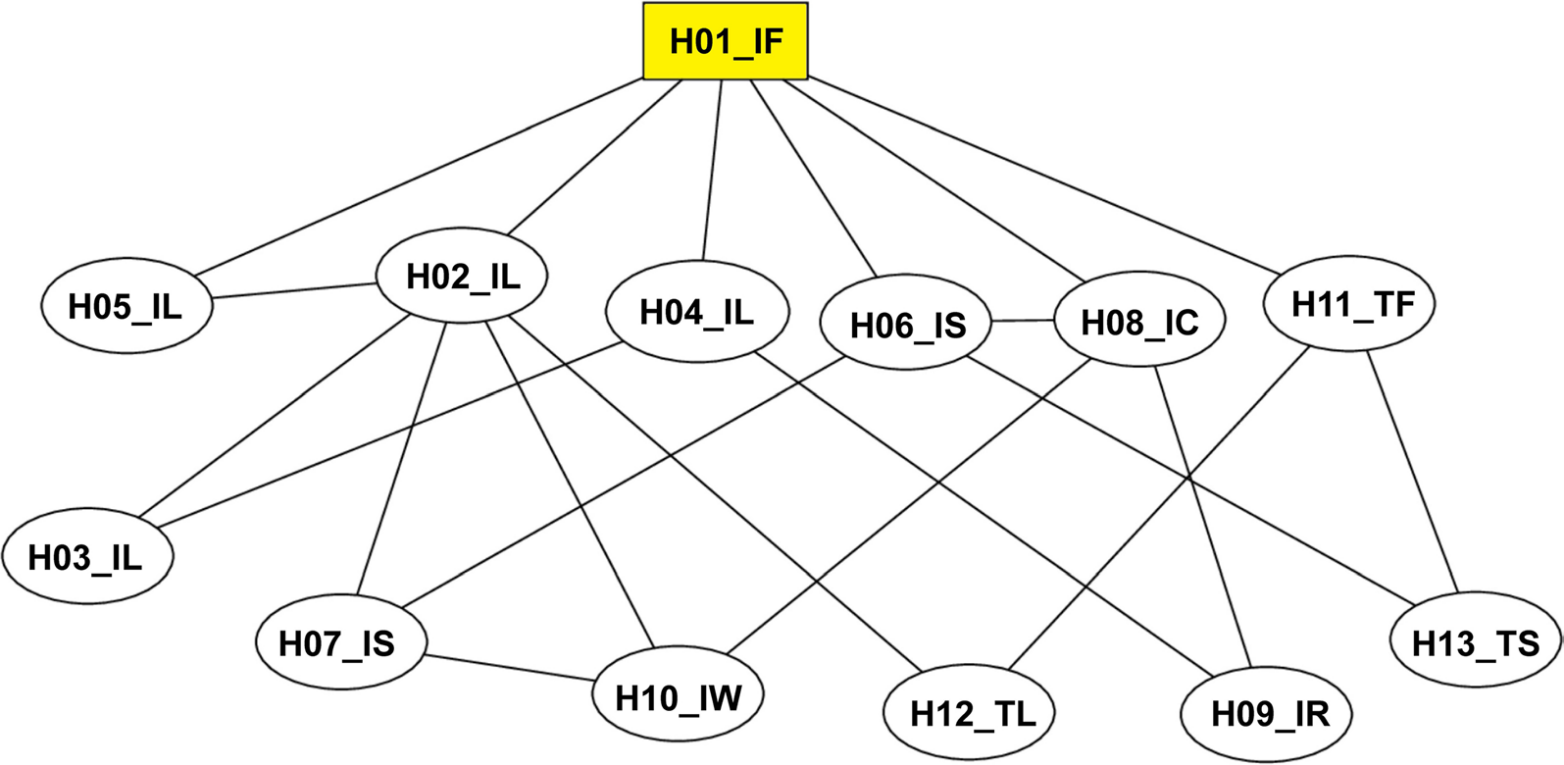
Genealogical relationships among haplotypes of *kdr* mutations of the *VGSC* gene estimated using TCS version 1.21 software. Each circle indicates one haplotype, and the number in each circle represents the haplotype type. The yellow square indicates the wild-type haplotype. Each unit branch represents one mutation

Based on the alleles in codons 1532 and 1534, 13 haplotypes were inferred and submitted to the NCBI (https://www.ncbi.nlm.nih.gov/) (Table 2). The 95% parsimony network showed a parallel evolutionary pathway. The ancestral wild-type haplotype (H01_IF) weight was only 0.13, which occurred in all populations. However, four haplotypes (H03_IL, H05_IL, H09_IR, and H10_IW) occurred in only one population. Six mutant haplotypes were formed by one-step mutation from ancestral haplotypes; these were H02_IL, H04_IL, H05_IL, H06_IS, H08_IC and H11_TF. The other six haplotypes were formed by one additional mutation (Fig. 3). This result supports the view that *kdr* mutations of *Ae. albopictus* have multiple origins.

**Table 2:** Haplotype of the voltage-gated sodium channel gene according to mutant alleles of codons 1532 and 1534 in *Ae. albopictus* populations from China

Haplotype	Allele		GenBank Accession number
	Codon 1532	Codon 1532	
H01_IF	I/ATC	F/TTC	MT559317
H02_IL	I/ATC	L/TTG	MT559318
H03_IL	I/ATC	L/CTG	MT559319
H04_IL	I/ATC	L/CTC	MT559320
H05_IL	I/ATC	L/TTA	MT559321
H06_IS	I/ATC	S/TCC	MT559322
H07_IS	I/ATC	S/TCG	MT559323
H08_IC	I/ATC	C/TGC	MT559324
H09_IR	I/ATC	R/CGC	MT559325
H10_IW	I/ATC	W/TGG	MT559326
H11_TF	T/ACC	F/TTC	MT559327
H12_TL	T/ACC	L/TTG	MT559328
H13_TS	T/ACC	S/TCC	MT559329

### Historical changes in *kdr* mutants in *Ae. albopictus* in China

No mutant allele was detected at codon 1532 of the *VGSC* gene in the 50 individuals collected in the 1990s. However, F1534S/TCC was found in the HNHK94 population with an unexpected frequency of 100% (Table 3). HNHK94 samples were collected from Haikou in 1994, 15 years earlier than the first reported *kdr* mutant populations by Kasai et al. [16]. Although the number of samples available is limited, the result showed that the F1534S *kdr* mutation of *Ae. albopictus* appeared no later than 1994 (HNHK94).

**Table 3:** Comparison of mutant allele frequency of the voltage-gated sodium channel gene in *Ae. albopictus* samples collected in the 1990s and 2010s

Population/time	Mutant allele frequency (%)		
	F1534S	F1534C	F1534L
GDGZ95/1990s	0	0	0
GDGZ/2010s	71.4	2.4	1.2
HNHK94/1990s	100.0	0	0
HNHK/2010s	61.9	4.0	0
SCCD97/1990s	0	0	0
GXNN/2010s^a^	43.5	39.8	3.7

^a^Because this study did not include the samples collected in the 2010s at the same collection point as SCCD97, we chose the GXNN population for comparison with the closest geographical distance

### Synonymous mutations

In addition to the abovem-entioned non-synonymous mutations, we found five synonymous mutations at codon 1516 CCG (P), 1528 TTC (F), 1539 ACC (T), 1540 CTC (L) and 1541 AAC (N) in the fragment. Only one mutant allele was detected at codons 1516 (CCA), 1528 (TTT) and 1541 (AAT), respectively, while two mutant alleles were found at codons 1539 (ACG, ACT) and 1540 (CTT, CTG). The frequencies of the mutant alleles were 20.8% (1516), 8.9% (1528), 3.8% (1539), 51.0% (1540) and 51.5% (1541) (Additional file 1: Table S6). However, no correlation was recorded between the synonymous and non-synonymous mutations at codons 1534 and 1532. The role of these synonymous mutations remains unexplored.

## Discussion

The use of pyrethroid insecticides began in China in the early 1970s, and they have been widely applied since the 1990s [38], with the use of pesticides in China increasing rapidly from 760,000 tons in 1991 to 1.8 million tons in 2011, at an average annual growth rate of 4.9% [39]. From 2006 to 2013, the demand for pyrethroid insecticides in mainland China was between 3600 tons and 8777 tons per year [39]. As sanitary insecticides, pyrethroids are mainly used as indoor sprays or in mosquito coils, or used to impregnate mosquito nets, drapes and screens. Many types of pyrethroids are used, including cypermethrin, fenvalerate, deltamethrin, beta-cypermethrin, tetramethrin, permethrin, methothrin, allethrin, resmethrin and cyhalothrin [38]. In Guangzhou, it is common to use multiple pyrethroid insecticides, with at least three kinds of pyrethroid insecticides applied in the same area [40]. In Hainan, 73.3–100.0% of the households use different pyrethroid insecticides for mosquito control [10].

However, with the widespread use of pyrethroid insecticides, insecticide resistance has become an important threat to the control of diseases transmitted by *Ae. albopictus*. In this study, we collected field populations of *Ae. albopictus* in different locations in China and detected and analyzed the *kdr* mutation to explore the characteristics and possible evolutionary trends of the *kdr* mutations. The sample collection sites in this study covered the main distribution range of *Ae. albopictus* in China from 16°N to 40°N [6], as well as tropical, subtropical and temperate climate zones.

Our results show that over the years different types of *kdr* mutations with a wide distribution in the field populations of *Ae. albopictus* have appeared in China. Certain populations in this study (SZSK) showed a high mutation rate of 1534 with complex mutation types. Two novel mutant alleles, F1534W/TGG and F1534R/CGC, were also detected in the SZSK population. The AAT of the collection site of this mosquito population was relatively high (23.0 ℃), allowing mosquitoes to reproduce all year round.

Moreover, increased development of trade and logistics, high population mobility and frequent use of insecticides in Shenzhen are all factors that promote insecticide resistance in mosquito populations [41]. If insecticides continue to be used in large quantities and indiscriminately, it is a real possibility that many field populations of *Ae. albopictus* will be found with a high frequency and multiple types of 1534 mutations in the future, similar to those in the SZSK population.

Unexpectedly, the F1534S mutation was detected in all 12 individuals of the HNHK94 population collected in Haikou City in 1994, indicating that even decades ago some populations of *Ae. albopictus* were highly resistant to insecticides. This result pushed the discovery time of *kdr* mutations in *Ae. albopictus* samples forward by 15 years [16]. This phenomenon may be related to the large-scale use of insecticides in Hainan Province after three dengue fever outbreaks in the last century: 1979–1982, 1985–1988 and 1991 [42]. The ZJHZ population was also collected after the occurrence of dengue fever in Hangzhou [11], which similarly showed a high percentage of 1534 mutation rates, suggesting that the large-scale use of insecticides is a factor that is closely related to the 1534 mutation rate of the *Ae. albopictus* population. Therefore, a more cautious, precise and strict insecticide resistance management (IRM) is urgently required [43]. Although the sample size is too small to allow any description of the overall pattern of *kdr* mutations in the *Ae. albopictus* population in the 1990s, the *kdr* mutation pattern seems to have become more complicated over time (Table 3).

According to the relative levels of the three indexes of the total rate of *kdr* mutation, F1534 mutation and I1532 mutation, there were two patterns observed in this study. One was high/high/low pattern, the other form was a low/low/high pattern. The first pattern was more likely to appear in areas with higher AAT, while the second was more likely mainly in areas with lower AAT. Moreover, the types of 1534 mutations were also more complex in the populations with the first pattern. There is an increasing body of evidence supporting a relationship between the F1534 and I1532 mutations and pyrethroid insecticide resistance. The F1534S and F1534C mutations have been found to be positively related to permethrin and deltamethrin resistance [12, 21], and the F1534S/L mutation reduces the channel sensitivity to type I pyrethroids but not to two type II pyrethroids [32]. The correlation between the I1532T mutation and insecticide resistance has been controversial [12, 32], however, our recent experimental results showed that this mutation was related to permethrin resistance, but not to deltamethrin resistance (unpublished data). In this study, the results show that the southern population in areas of higher AAT was more resistant to insecticides than the northern population in areas of relatively lower AAT. The mosquitoes in the areas of higher AAT have more generations and can therefore be regarded as fast-evolving populations. In contrast, the populations in the areas of lower AAT can be regarded as slow-evolving populations. Our results suggest that the highly resistant population may be screened out within a short period in the fast-evolving populations. Due to the long cold winter in northern China, insecticides are often used seasonally, which may slow down the development of insecticide resistance, consequently suggesting that seasonal use of insecticides may be one strategy to slow down the development of insecticide resistance. However, using insecticides seasonally in a certain area and effectively controlling the risk of vector-borne disease transmission requires further research. Such an approach is crucial for preventing and controlling mosquito-borne diseases in the context of global climate change [44].

In our study, the *kdr* mutation pattern of the *Ae. albopictus* population was found to correlate with AAT and AMT in January; however, this does not mean that the temperature is the direct driving factor for the *kdr* mutation evolution. In areas with higher temperatures, the populations of *Ae. albopictus* reproduce faster, with more generations yearly, and insecticides are continuously applied throughout the year to control the risk of mosquito-borne diseases; these two factors accelerate evolution of the *kdr* mutation in field populations of *Ae. albopictus*. This phenomenon reminds us that with the use of insecticides, especially in the areas with higher temperatures, we need to pay more attention to delaying the development of pesticide resistance.

Although some meaningful results were obtained in our study, there are still some limitations. First, only a few historical samples are available, making it impossible to analyze the historical changes of *kdr* mutations systematically. Also, accurate information on the use of insecticides cannot be collected, which affected the interpretation of the results. Moreover, this study focused on the most common *kdr* mutations in *Ae. albopictus*, leaving some types of *kdr* mutations with a low mutation rate unstudied [18]. Many different mosquito collection methods were used in this study, and the collection period was long, which may affect the accuracy of the results. More rigorous sample collection methods could help improve the accuracy of research results. Finally, although the synonymous mutations and intron length polymorphisms have been documented previously in populations of *Ae. albopictus* and *Ae. aegypti* [19, 28, 45], the significance of the synonymous mutations found in this study is unclear, and further research is needed.

## Conclusions

In the present study, we revealed that in China, *kdr* mutations are widespread in the field populations of *Ae. albopictus*. Two novel mutant alleles, namely F1534W/TGG and F1534R/CGC, were detected for the first time. The 1534 *kdr* mutation appeared in the population of *Ae. albopictus* no later than 1994. HNHK94 was the earliest known *Ae. albopictus* field population with a *kdr* mutation. The F1534 mutation rate was found to be positively correlated with AAT, while the I1532 mutation rate was negatively correlated with AAT. Insecticide usage should be carefully managed to slow down the spread of highly resistant *Ae. albopictus* populations, especially in the areas with higher AAT.

## Supplementary Information

**Additional file1:Table S1.** Sampling information on* Aedes albopictus* field populations in China. **Table S2.** The annual average temperature (AAT) of collection sites. **Table S3.** The allele frequency at codons 1532 and 1534 of the* VGSC* gene in each* Ae. albopictus* population from China. **Table S4.** The frequency of the* VGSC* gene at codon 1534 genotypes in* Ae. albopictus* samples from China. **Table S5.** The frequency of the* VGSC* genotype at codon 1532 in* Ae. albopictus* samples from China. **Table S6.** The synonymous mutation allele frequency of the* VGSC* gene in* Ae. albopictus* samples from China.

## Data Availability

The datasets used and/or analyzed during the present study are available from the corresponding authors on reasonable request, and sequences are available in GenBank (accession numbers: MT559317, MT559318, MT559319, MT559320, MT559321, MT559322, MT559323, MT559324, MT559325, MT559326, MT559327, MT559328, and MT559329).

## Abbreviations

AAT: Annual average temperature
IRM: Insecticide resistance management
*kdr*: Knockdown resistance
*VGSC*: Voltage-gated sodium channel gene
