# Supplementary material for: The pattern of kdr mutations correlated with the temperature in field populations of Aedes albopictus in China
Source: Parasit Vectors. 2021 Aug 16;14:406. doi: 10.1186/s13071-021-04906-z (PMC8365938; doi:10.1186/s13071-021-04906-z)
Supplement: Supplementary file 1 — Additional file1:Table S1. Sampling information on Aedes albopictus field populations in China. Table S2. The annual average temperature (AAT) of collection sites. Table S3. The allele frequency at codons 1532 and 1534 of the VGSC gene in each Ae. albopictus population from China. Table S4. The frequency of the VGSC gene at codon 1534 genotypes in Ae. albopictus samples from China. Table S5. The frequency of the VGSC genotype at codon 1532 in Ae. albopictus samples from China. Table S6. The synonymous mutation allele frequency of the VGSC gene in Ae. albopictus samples from China. [file 13071_2021_4906_MOESM1_ESM.docx]

**Additional file 1: Table S1.** Table 1 Sampling information of *Aedes albopictus* field populations in China

| Collection sites | | | Population | | Latitude/longitude coordinates | Sampling environment | Date | Collecting method/stages | | Breeding sites | |
| --- | --- | --- | --- | --- | --- | --- | --- | --- | --- | --- | --- |
| Beijing | Fengtai | BJFT | | 39°51′ N/116°16′ E | | Residential area, urban | July 2018 | Sucking tube/adult | 7 | |  |
| Guangdong | Guangzhou | GDGZ95 ^b^ | | 23°07′ N/113°15′ E | | Outdoor area of office building, urban | June 1995 | Scooping/larvae, pupae | 5 | |  |
|  |  | GDGZ | |  |  |  | November 2019 | Scooping/larvae, pupae | 10 | |  |
|  | Shantou | GDST | | 23°21′ N/116°40′ E | | Park, suburban | May 2018 | Scooping/larvae | 12 | |  |
|  | Shenzhen | SZSK | | 22°29′ N/113°54′ E | | Flower nursery, urban | June 2018 | Scooping/larvae, pupae | 11 | |  |
| Guangxi | Nanning | GXNN | | 22°47′ N/108°19′ E | | Kindergarten, urban | May 2020 | Scooping/larvae | 10 | |  |
| Hainan | Haikou | HNHK94 ^b^ | | 20°02′ N/110°11′ E | | Residential area, suburban, island | June 1994 | Scooping/larvae, pupae | 5 | |  |
|  |  | HNHK ^a^ | |  |  |  | June-October 2017 | Scooping/larvae, pupae | 16 | |  |
|  | Yongxing Island | HNSS | | 16°49′ N/112°20′ E | | urban, tropic island | April 2019 | BG-trap/adult | - | |  |
|  | Sanya | HNSY | | 18°15′ N/109°30′ E | | Village, island | June 2019 | Scooping/larvae, pupae | 12 | |  |
| Jiangsu | Nanjing | JSNJ | | 32°03′ N/118°47′ E | | Park, urban | October 2017 | Scooping/larvae | 9 | |  |
| Sichuan | Chengdu | SCCD97 ^b^ | | 30°34′ N/104°03′ E | | Park, suburban | June 1997 | Scooping/larvae, pupae | 6 | |  |
| Shandong | Jinan | SDJNan | | 36°39′ N/117°06′ E | | Park, urban | August 2017 | Scooping/larvae, pupae | 18 | |  |
|  | Jining | SDJNing | | 35°24′ N/116°34′ E | | Village | July 2018 | Scooping/larvae, pupae | 10 | |  |
| Shanghai  (SH) | Baoshan | SHBS ^a^ | | 31°24′ N/121°29′ E | | Residential area, suburban | August 2017 | Scooping/larvae | 12 | |  |
|  | Yangpu Park | SHGQ | | 31°19′ N/121°32′ E | | Park, urban | September-October 2017 | Scooping/larvae | 20 | |  |
|  | Yangpu | SHYP ^a^ | | 31°15′ N/121°31′ E | | Outdoor area of office building, urban | June-October 2017 | BG-trap/adult | - | |  |
| Shanxi  (SX) | Yangquan | SX | | 37°51′ N/113°34′ E | | Residential area, urban | June-October 2017 | Scooping/larvae | 6 | |  |
|  | Yuncheng |  |  | 35°01′ N/111°0′ E | |  |  | Scooping/larvae | 6 | |  |
| Shaanxi | Xian | SXXA | | 34°20′ N/108°56′ E | | Residential area, urban | September-October 2017 | Light trap/adult | - | |  |
|  |  |  |  |  |  |  | June-October 2018 | Light trap/adult | - | |  |
| Yunnan | Jinghong | YNJH | | 22°02′ N/100°47′ E | | Outdoor, urban | October 2016 | Scooping/larvae, pupae | 10 | |  |
|  |  |  |  |  |  |  | September 2017 | Sucking tube/adult | - | |  |
| Zhejiang | Hangzhou | ZJHZ ^a^ | | 30°14′ N/120°12′ E | | Residential area, urban; surrounding area of restaurant, suburban | September 2017 | Scooping/larvae; sucking tube/adult | 8 | |  |

^a^ The *kdr* mutations information of these populations has been used to analyses in our previous published article [12, 30]

^b^ The field populations collected in 1990s. -, no larvae or pupae was collected.

**Additional file 1: Table S2.** The annual average temperature (AAT) of collection sites

| Population | Province | City | Weather station | Station number | Valid period | AAT(℃) | AMT in January (℃) | AMT in July (℃) |
| --- | --- | --- | --- | --- | --- | --- | --- | --- |
| BJFT | Beijing | Fengtai | Fengtai | 54514 | 2010~2019 | 13.5 | -3.0 | 27.7 |
| GDGZ | Guangdong | Guangzhou | Guangzhou | 59287 | 2010~2019 | 22.0 | 13.3 | 28.5 |
| GDST | Guangdong | Shantou | Shantou | 59316 | 2010~2019 | 23.0 | 14.9 | 29.5 |
| SZSK | Guangdong | Shenzhen | Shenzhen | 59493 | 2010~2019 | 23.3 | 15.6 | 28.9 |
| GXNN | Guangxi | Nanning | Nanning | 59431 | 2010~2019 | 21.7 | 12.4 | 28.2 |
| HNHK | Hainan | Haikou | Haikou | 59758 | 2010~2019 | 24.6 | 17.8 | 28.6 |
| HNSS | Hainan | Sansha | Dongfang | 59838 | 2010~2019 | 25.7 | 19.5 | 29.4 |
| HNSY | Hainan | Sanya | Dongfang | 59838 | 2010~2019 | 25.7 | 19.5 | 29.4 |
| JSNJ | Jiangsu | Nanjing | Nanjing | 58238 | 2010~2019 | 16.6 | 3.5 | 28.7 |
| SDJNan | Shandong | Jinan | Jinan | 54823 | 2010~2019 | 15.0 | -0.3 | 28.0 |
| SDJNing | Shandong | Jining | Yanzhou | 54916 | 2010~2019 | 14.3 | -0.8 | 26.9 |
| SHBS | Shanghai | Baoshan | Baoshan | 58362 | 2010~2019 | 17.3 | 5.0 | 29.3 |
| SHGQ | Shanghai | Yangpu | Baoshan | 58362 | 2010~2019 | 17.3 | 5.0 | 29.3 |
| SHYP | Shanghai | Yangpu | Baoshan | 58362 | 2010~2019 | 17.3 | 5.0 | 29.3 |
| SX | Shanxi | Yangquan | Yangquan | 53782 | 2010~2019 | 11.1 | -3.9 | 24.1 |
| SXXA | Shaanxi | Xian | Wugong | 57034 | 2010~2019 | 13.8 | -0.4 | 26.9 |
| YNJH | Yunnan | Jinghong | Simao | 56964 | 2010~2019 | 19.7 | 13.9 | 22.7 |
| ZJHZ | Zhejiang | Hangzhou | Hangzhou | 58457 | 2010~2019 | 17.7 | 5.2 | 29.7 |

Abbreviations: AAT, annual average temperature; AMT, average monthly temperature.

**Additional file 1: Table S3.** The allele frequency at codon 1532 and 1534 of *VGSC* gene in each *Aedes albopictus* population from China

| Sample code | Sample size | | Codon 1532 | | |  | Codon 1534 | | | | | | | | | | | | | |
| --- | --- | --- | --- | --- | --- | --- | --- | --- | --- | --- | --- | --- | --- | --- | --- | --- | --- | --- | --- | --- |
|  |  |  | Wildtype |  | Mutant |  | Wildtype |  | Mutant | | | | | | | | | | | |
|  |  |  | ATC/I |  | ACC/T |  | TTC/F |  | TTG/L | CTG/L | | CTC/L | | TTA/L | TCC/S | TCG/S | | TGC/C | CGC/R | TGG/W |
| BJFT | 58 | *N* | 73 |  | 43 |  | 115 |  | 0 | 0 | | 0 | | 0 | 1 | 0 | | 0 | 0 | 0 |
|  |  | *P* (%) | 62.93 |  | 37.07 |  | 99.14 |  | 0 | 0 | | 0 | | 0 | 0.86 | 0 | | 0 | 0 | 0 |
| GDGZ | 42 | *N* | 84 |  | 0 |  | 21 |  | 1 | 0 | | 0 | | 0 | 60 | 0 | | 2 | 0 | 0 |
|  |  | *P* (%) | 100.00 |  | 0 |  | 25.00 |  | 1.19 | 0 | | 0 | | 0 | 71.43 | 0 | | 2.38 | 0 | 0 |
| GDST | 60 | *N* | 120 |  | 0 |  | 120 |  | 0 | 0 | | 0 | | 0 | 0 | 0 | | 0 | 0 | 0 |
|  |  | *P* (%) | 100.00 |  | 0 |  | 100.00 |  | 0 | 0 | | 0 | | 0 | 0 | 0 | | 0 | 0 | 0 |
| SZSK | 60 | *N* | 120 |  | 0 |  | 37 |  | 26 | 4 | | 10 | | 0 | 9 | 2 | | 21 | 8 | 3 |
|  |  | *P* (%) | 100.00 |  | 0 |  | 30.83 |  | 21.67 | 3.33 | | 8.33 | | 0 | 7.50 | 1.67 | | 17.50 | 6.67 | 2.50 |
| GXNN | 54 | *N* | 108 |  | 0 |  | 16 |  | 1 | 0 | | 1 | | 0 | 47 | 0 | | 43 | 0 | 0 |
|  |  | *P* (%) | 100.00 |  | 0 |  | 14.81 |  | 0.93 | 0 | | 0.93 | | 0 | 43.52 | 0 | | 39.81 | 0 | 0 |
| HNHK | 139 | *N* | 278 |  | 0 |  | 95 |  | 0 | 0 | | 0 | | 0 | 172 | 0 | | 11 | 0 | 0 |
|  |  | *P* (%) | 100.00 |  | 0 |  | 34.17 |  | 0 | 0 | | 0 | | 0 | 61.87 | 0 | | 3.96 | 0 | 0 |
| HNSS | 36 | *N* | 72 |  | 0 |  | 26 |  | 0 | 0 | | 0 | | 0 | 46 | 0 | | 0 | 0 | 0 |
|  |  | *P* (%) | 100.00 |  | 0 |  | 36.11 |  | 0 | 0 | | 0 | | 0 | 63.89 | 0 | | 0 | 0 | 0 |
| HNSY | 91 | *N* | 182 |  | 0 |  | 119 |  | 42 | 0 | | 1 | | 0 | 18 | 0 | | 2 | 0 | 0 |
|  |  | *P* (%) | 100.00 |  | 0 |  | 65.38 |  | 23.08 | 0 | | 0.55 | | 0 | 9.89 | 0 | | 1.10 | 0 | 0 |
| JSNJ | 47 | *N* | 87 |  | 7 |  | 90 |  | 0 | 0 | | 0 | | 0 | 4 | 0 | | 0 | 0 | 0 |
|  |  | *P* (%) | 92.55 |  | 7.45 |  | 95.74 |  | 0 | 0 | | 0 | | 0 | 4.26 | 0 | | 0 | 0 | 0 |
| SDJNan | 108 | *N* | 216 |  | 0 |  | 190 |  | 0 | 0 | | 0 | | 23 | 3 | 0 | | 0 | 0 | 0 |
|  |  | *P* (%) | 100.00 |  | 0 |  | 87.96 |  | 0 | 0 | | 0 | | 10.65 | 1.39 | 0 | | 0 | 0 | 0 |
| SDJNing | 44 | *N* | 81 |  | 7 |  | 88 |  | 0 | 0 | | 0 | | 0 | 0 | 0 | | 0 | 0 | 0 |
|  |  | *P* (%) | 92.05 |  | 7.95 |  | 100.00 |  | 0 | 0 | | 0 | | 0 | 0 | 0 | | 0 | 0 | 0 |
| SHBS | 138 | *N* | 253 |  | 23 |  | 214 |  | 0 | 0 | | 0 | | 0 | 62 | 0 | | 0 | 0 | 0 |
|  |  | *P* (%) | 91.67 |  | 8.33 |  | 77.54 |  | 0 | 0 | | 0 | | 0 | 22.46 | 0 | | 0 | 0 | 0 |
| SHGQ | 126 | *N* | 207 |  | 45 |  | 159 |  | 0 | 0 | | 0 | | 0 | 93 | 0 | | 0 | 0 | 0 |
|  |  | *P* (%) | 82.14 |  | 17.86 |  | 63.10 |  | 0 | 0 | | 0 | | 0 | 36.90 | 0 | | 0 | 0 | 0 |
| SHYP | 98 | *N* | 161 |  | 35 |  | 102 |  | 2 | 0 | | 0 | | 0 | 91 | 1 | | 0 | 0 | 0 |
|  |  | *P* (%) | 82.14 |  | 17.86 |  | 52.04 |  | 1.02 | 0 | | 0 | | 0 | 46.43 | 0.51 | | 0 | 0 | 0 |
| SX | 62 | *N* | 115 |  | 9 |  | 123 |  | 0 | 0 | | 0 | | 0 | 1 | 0 | | 0 | 0 | 0 |
|  |  | *P* (%) | 92.74 |  | 7.26 |  | 99.19 |  | 0 | 0 | | 0 | | 0 | 0.81 | 0 | | 0 | 0 | 0 |
| SXXA | 25 | *N* | 32 |  | 18 |  | 50 |  | 0 | 0 | | 0 | | 0 | 0 | 0 | | 0 | 0 | 0 |
|  |  | *P* (%) | 64.00 |  | 36.00 |  | 100.00 |  | 0 | 0 | | 0 | | 0 | 0 | 0 | | 0 | 0 | 0 |
| YNJH | 77 | *N* | 144 |  | 10 |  | 72 |  | 4 | 0 | | 0 | | 0 | 73 | 1 | | 4 | 0 | 0 |
|  |  | *P* (%) | 93.51 |  | 6.49 |  | 46.75 |  | 2.60 | 0 | | 0 | | 0 | 47.40 | 0.65 | | 2.60 | 0 | 0 |
| ZJHZ | 284 | *N* | 567 |  | 1 |  | 48 |  | 0 | 0 | | 0 | | 0 | 520 | 0 | | 0 | 0 | 0 |
|  |  | *P* (%) | 99.82 |  | 0.18 |  | 8.45 |  | 0 | 0 | | 0 | | 0 | 91.55 | 0 | | 0 | 0 | 0 |
| Total | 1549 | *N* | 2900 |  | 198 |  | 1685 |  | 76 | 4 | | 12 | | 23 | 1200 | 4 | | 83 | 8 | 3 |
|  |  | *P* (%) | 93.61 |  | 6.39 |  | 54.39 |  | 2.45 | 0.13 | 0.39 | | 0.74 | | 38.73 | | 0.13 | 2.68 | 0.26 | 0.10 |

Abbreviations: *N*, number of the allele; *P*, frequency of the allele.

**Additional file 1: Table S4.** The frequency of *VGSC* genotype at codon 1534 in *Aedes albopictus* samples from China

| Population | Sample size | | Wildtype genotype |  | Wildtype/mutant heterozygote | | | |  | Mutant genotype | | | | | | | | | |
| --- | --- | --- | --- | --- | --- | --- | --- | --- | --- | --- | --- | --- | --- | --- | --- | --- | --- | --- | --- |
|  |  |  | F/F |  | F/L | F/S | F/C | F/R |  | L/L | C/C | S/S | C/R | L/C | L/R | C/W | S/R | L/S | S/C |
| YNJH | 77 | *N* | 21 |  | 3 | 25 | 2 | 0 |  | 0 | 1 | 24 | 0 | 0 | 0 | 0 | 0 | 1 | 0 |
|  |  | *P* (%) | 27.27 |  | 3.89 | 32.47 | 2.60 | 0 |  | 0 | 1.30 | 31.17 | 0 | 0 | 0 | 0 | 0 | 1.30 | 0 |
| SHBS | 138 | *N* | 85 |  | 0 | 44 | 0 | 0 |  | 0 | 0 | 9 | 0 | 0 | 0 | 0 | 0 | 0 | 0 |
|  |  | *P* (%) | 61.59 |  | 0 | 31.89 | 0 | 0 |  | 0 | 0 | 6.52 | 0 | 0 | 0 | 0 | 0 | 0 | 0 |
| SHYP | 98 | *N* | 27 |  | 2 | 46 | 0 | 0 |  | 0 | 0 | 23 | 0 | 0 | 0 | 0 | 0 | 0 | 0 |
|  |  | *P* (%) | 27.55 |  | 2.04 | 46.94 | 0 | 0 |  | 0 | 0 | 23.47 | 0 | 0 | 0 | 0 | 0 | 0 | 0 |
| SHGQ | 126 | *N* | 51 |  | 0 | 57 | 0 | 0 |  | 0 | 0 | 18 | 0 | 0 | 0 | 0 | 0 | 0 | 0 |
|  |  | *P* (%) | 40.48 |  | 0 | 45.24 | 0 | 0 |  | 0 | 0 | 14.28 | 0 | 0 | 0 | 0 | 0 | 0 | 0 |
| ZJHZ | 284 | *N* | 3 |  | 0 | 42 | 0 | 0 |  | 0 | 0 | 239 | 0 | 0 | 0 | 0 | 0 | 0 | 0 |
|  |  | *P* (%) | 1.06 |  | 0 | 14.79 | 0 | 0 |  | 0 | 0 | 84.15 | 0 | 0 | 0 | 0 | 0 | 0 | 0 |
| SXXA | 25 | *N* | 25 |  | 0 | 0 | 0 | 0 |  | 0 | 0 | 0 | 0 | 0 | 0 | 0 | 0 | 0 | 0 |
|  |  | *P* (%) | 100.00 |  | 0 | 0 | 0 | 0 |  | 0 | 0 | 0 | 0 | 0 | 0 | 0 | 0 | 0 | 0 |
| JSNJ | 47 | *N* | 43 |  | 0 | 4 | 0 | 0 |  | 0 | 0 | 0 | 0 | 0 | 0 | 0 | 0 | 0 | 0 |
|  |  | *P* (%) | 91.49 |  | 0 | 8.51 | 0 | 0 |  | 0 | 0 | 0 | 0 | 0 | 0 | 0 | 0 | 0 | 0 |
| SX | 62 | *N* | 61 |  | 0 | 1 | 0 | 0 |  | 0 | 0 | 0 | 0 | 0 | 0 | 0 | 0 | 0 | 0 |
|  |  | *P* (%) | 98.39 |  | 0 | 1.61 | 0 | 0 |  | 0 | 0 | 0 | 0 | 0 | 0 | 0 | 0 | 0 | 0 |
| HNHK | 139 | *N* | 14 |  | 0 | 56 | 11 | 0 |  | 0 | 0 | 58 | 0 | 0 | 0 | 0 | 0 | 0 | 0 |
|  |  | *P* (%) | 10.07 |  | 0 | 40.29 | 7.91 | 0 |  | 0 | 0 | 41.73 | 0 | 0 | 0 | 0 | 0 | 0 | 0 |
| GDST | 60 | *N* | 60 |  | 0 | 0 | 0 | 0 |  | 0 | 0 | 0 | 0 | 0 | 0 | 0 | 0 | 0 | 0 |
|  |  | *P* (%) | 100.00 |  | 0 | 0 | 0 | 0 |  | 0 | 0 | 0 | 0 | 0 | 0 | 0 | 0 | 0 | 0 |
| SZSK | 60 | *N* | 4 |  | 16 | 5 | 7 | 1 |  | 9 | 2 | 2 | 4 | 3 | 2 | 3 | 1 | 1 | 0 |
|  |  | *P* (%) | 6.67 |  | 26.66 | 8.33 | 11.67 | 1.67 |  | 15.00 | 3.33 | 3.33 | 6.67 | 5.00 | 3.33 | 5.00 | 1.67 | 1.67 | 0 |
| BJFT | 58 | *N* | 57 |  | 0 | 1 | 0 | 0 |  | 0 | 0 | 0 | 0 | 0 | 0 | 0 | 0 | 0 | 0 |
|  |  | *P* (%) | 98.28 |  | 0 | 1.72 | 0 | 0 |  | 0 | 0 | 0 | 0 | 0 | 0 | 0 | 0 | 0 | 0 |
| SDJNan | 108 | *N* | 86 |  | 15 | 3 | 0 | 0 |  | 4 | 0 | 0 | 0 | 0 | 0 | 0 | 0 | 0 | 0 |
|  |  | *P* (%) | 79.63 |  | 13.89 | 2.78 | 0 | 0 |  | 3.70 | 0 | 0 | 0 | 0 | 0 | 0 | 0 | 0 | 0 |
| SDJNing | 44 | *N* | 44 |  | 0 | 0 | 0 | 0 |  | 0 | 0 | 0 | 0 | 0 | 0 | 0 | 0 | 0 | 0 |
|  |  | *P* (%) | 100.00 |  | 0 | 0 | 0 | 0 |  | 0 | 0 | 0 | 0 | 0 | 0 | 0 | 0 | 0 | 0 |
| HNSS | 36 | *N* | 4 |  | 0 | 18 | 0 | 0 |  | 0 | 0 | 14 | 0 | 0 | 0 | 0 | 0 | 0 | 0 |
|  |  | *P* (%) | 11.11 |  | 0 | 50.00 | 0 | 0 |  | 0 | 0 | 38.89 | 0 | 0 | 0 | 0 | 0 | 0 | 0 |
| HNSY | 91 | *N* | 40 |  | 23 | 14 | 2 | 0 |  | 10 | 0 | 2 | 0 | 0 | 0 | 0 | 0 | 0 | 0 |
|  |  | *P* (%) | 43.96 |  | 25.27 | 15.38 | 2.20 | 0 |  | 10.99 | 0 | 2.20 | 0 | 0 | 0 | 0 | 0 | 0 | 0 |
| GDGZ | 42 | *N* | 2 |  | 1 | 15 | 1 | 0 |  | 0 | 0 | 22 | 0 | 0 | 0 | 0 | 0 | 0 | 1 |
|  |  | *P* (%) | 4.76 |  | 2.38 | 35.72 | 2.38 | 0 |  | 0 | 0 | 52.38 | 0 | 0 | 0 | 0 | 0 | 0 | 2.38 |
| GXNN | 54 | *N* | 2 |  | 2 | 7 | 3 | 0 |  | 0 | 9 | 6 | 0 | 0 | 0 | 0 | 0 | 0 | 25 |
|  |  | *P* (%) | 3.70 |  | 3.70 | 12.96 | 5.56 | 0 |  | 0 | 16.67 | 11.11 | 0 | 0 | 0 | 0 | 0 | 0 | 46.30 |
| Total | 1549 | *N* | 629 |  | 62 | 338 | 26 | 1 |  | 23 | 12 | 417 | 4 | 3 | 2 | 3 | 1 | 2 | 26 |
|  |  | *P* (%) | 40.61 |  | 4.01 | 21.82 | 1.68 | 0.06 |  | 1.48 | 0.77 | 26.93 | 0.26 | 0.19 | 0.13 | 0.19 | 0.06 | 0.13 | 1.68 |

*N*: number of the genotype; *P*: frequency of the genotype.

**Additional file 1: Table S5.** The frequency of *VGSC* gene at codon 1532 genotypes in *Aedes albopictus* samples from China

| Population | Sample size | | Wildtype genotype | Wildtype/mutant heterozygote | Mutant genotype |
| --- | --- | --- | --- | --- | --- |
|  |  |  | I/I | I/T | T/T |
| YNJH | 77 | *N* | 67 | 10 | 0 |
|  |  | *P* (%) | 87.01 | 12.99 | 0 |
| SHBS | 138 | *N* | 116 | 21 | 1 |
|  |  | *P* (%) | 84.06 | 15.22 | 0.72 |
| SHYP | 98 | *N* | 68 | 25 | 5 |
|  |  | *P* (%) | 69.39 | 25.51 | 5.10 |
| SHGQ | 126 | *N* | 84 | 39 | 3 |
|  |  | *P* (%) | 66.67 | 30.95 | 2.38 |
| ZJHZ | 284 | *N* | 283 | 1 | 0 |
|  |  | *P* (%) | 99.65 | 0.35 | 0 |
| SXXA | 25 | *N* | 10 | 12 | 3 |
|  |  | *P* (%) | 40.00 | 48.00 | 12.00 |
| JSNJ | 47 | *N* | 40 | 7 | 0 |
|  |  | *P* (%) | 85.11 | 14.89 | 0 |
| SX | 62 | *N* | 53 | 9 | 0 |
|  |  | *P* (%) | 85.48 | 14.52 | 0 |
| HNHK | 139 | *N* | 139 | 0 | 0 |
|  |  | *P* (%) | 100.00 | 0 | 0 |
| GDST | 60 | *N* | 60 | 0 | 0 |
|  |  | *P* (%) | 100.00 | 0 | 0 |
| SZSK | 60 | *N* | 60 | 0 | 0 |
|  |  | *P* (%) | 100.00 | 0 | 0 |
| BJFT | 58 | *N* | 23 | 27 | 8 |
|  |  | *P* (%) | 39.66 | 46.55 | 13.79 |
| SDJNan | 108 | *N* | 108 | 0 | 0 |
|  |  | *P* (%) | 100.00 | 0 | 0 |
| SDJNing | 44 | *N* | 38 | 5 | 1 |
|  |  | *P* (%) | 86.36 | 11.37 | 2.27 |
| HNSS | 36 | *N* | 36 | 0 | 0 |
|  |  | *P* (%) | 100.00 | 0 | 0 |
| HNSY | 91 | *N* | 91 | 0 | 0 |
|  |  | *P* (%) | 100.00 | 0 | 0 |
| GDGZ | 42 | *N* | 42 | 0 | 0 |
|  |  | *P* (%) | 100.00 | 0 | 0 |
| GXNN | 54 | *N* | 54 | 0 | 0 |
|  |  | *P* (%) | 100.00 | 0 | 0 |
| Total | 1549 | *N* | 1372 | 156 | 21 |
|  |  | *P* (%) | 88.57 | 10.07 | 1.36 |

*N*: number of the genotype; *P*: frequency of the genotype.

**Additional file 1: Table S6.** The synonymous mutation allele frequency of *VGSC* gene in *Aedes albopictus* samples from China

| Population | Sample size | | Codon 1516 | | |  | Codon 1528 | |  | Codon 1539 | | |  | Codon 1540 | | |  | Codon 1541 | |
| --- | --- | --- | --- | --- | --- | --- | --- | --- | --- | --- | --- | --- | --- | --- | --- | --- | --- | --- | --- |
|  |  |  | Wildtype | Mutant | |  | Wildtype | Mutant |  | Wildtype | Mutant | |  | Wildtype | Mutant | |  | Wildtype | Mutant |
|  |  |  | CCG/P | CCA/P | CCC/P |  | TTC/F | TTT/F |  | ACC/T | ACG/T | ACT/T |  | CTC/L | CTT/ L | CTG/ L |  | AAC/N | AAT/N |
| YNJH | 77 | *N* | 75 | 79 | 0 |  | 148 | 6 |  | 148 | 6 | 0 |  | 54 | 90 | 10 |  | 59 | 95 |
|  |  | *P* (%) | 48.70 | 51.30 | 0 |  | 96.10 | 3.90 |  | 96.10 | 3.90 | 0 |  | 35.07 | 58.44 | 6.49 |  | 38.31 | 61.69 |
| SHBS | 138 | *N* | 227 | 49 | 0 |  | 265 | 11 |  | 271 | 0 | 5 |  | 149 | 127 | 0 |  | 148 | 128 |
|  |  | *P* (%) | 82.25 | 17.75 | 0 |  | 96.01 | 3.99 |  | 98.19 | 0 | 1.81 |  | 53.99 | 46.01 | 0 |  | 53.62 | 46.38 |
| SHYP | 98 | *N* | 191 | 5 | 0 |  | 194 | 2 |  | 192 | 0 | 4 |  | 48 | 148 | 0 |  | 49 | 147 |
|  |  | *P* (%) | 97.45 | 2.55 | 0 |  | 98.98 | 1.02 |  | 97.96 | 0 | 2.04 |  | 24.49 | 75.51 | 0 |  | 25.00 | 75.00 |
| SHGQ | 126 | *N* | 230 | 22 | 0 |  | 245 | 7 |  | 252 | 0 | 0 |  | 163 | 89 | 0 |  | 163 | 89 |
|  |  | *P* (%) | 91.27 | 8.73 | 0 |  | 97.22 | 2.78 |  | 100.00 | 0 | 0 |  | 64.68 | 35.32 | 0 |  | 64.68 | 35.32 |
| ZJHZ | 284 | *N* | 564 | 4 | 0 |  | 563 | 5 |  | 568 | 0 | 0 |  | 44 | 520 | 4 |  | 45 | 523 |
|  |  | *P* (%) | 99.30 | 0.70 | 0 |  | 99.12 | 0.88 |  | 100.00 | 0 | 0 |  | 7.75 | 91.55 | 0.70 |  | 7.92 | 92.08 |
| SXXA | 25 | *N* | 45 | 5 | 0 |  | 50 | 0 |  | 50 | 0 | 0 |  | 26 | 24 | 0 |  | 24 | 26 |
|  |  | *P* (%) | 90.00 | 10.00 | 0 |  | 100.00 | 0 |  | 100.00 | 0 | 0 |  | 52.00 | 48.00 | 0 |  | 48.00 | 52.00 |
| JSNJ | 47 | *N* | 80 | 14 | 0 |  | 78 | 16 |  | 94 | 0 | 0 |  | 63 | 31 | 0 |  | 64 | 30 |
|  |  | *P* (%) | 85.11 | 14.89 | 0 |  | 82.98 | 17.02 |  | 100.00 | 0 | 0 |  | 67.02 | 32.98 | 0 |  | 68.09 | 31.91 |
| SX | 62 | *N* | 75 | 49 | 0 |  | 120 | 4 |  | 123 | 0 | 1 |  | 101 | 19 | 4 |  | 94 | 30 |
|  |  | *P* (%) | 60.48 | 39.52 | 0 |  | 96.77 | 3.23 |  | 99.19 | 0 | 0.81 |  | 81.45 | 15.32 | 3.23 |  | 75.81 | 24.19 |
| HNHK | 139 | *N* | 96 | 182 | 0 |  | 243 | 35 |  | 277 | 0 | 1 |  | 60 | 218 | 0 |  | 60 | 218 |
|  |  | *P* (%) | 34.53 | 65.47 | 0 |  | 87.41 | 12.59 |  | 99.64 | 0 | 0.36 |  | 21.58 | 78.42 | 0 |  | 21.58 | 78.42 |
| GDST | 60 | *N* | 113 | 7 | 0 |  | 116 | 4 |  | 120 | 0 | 0 |  | 115 | 5 | 0 |  | 109 | 11 |
|  |  | *P* (%) | 94.17 | 5.83 | 0 |  | 96.67 | 3.33 |  | 100.00 | 0 | 0 |  | 95.83 | 4.17 | 0 |  | 90.83 | 9.17 |
| SZSK | 60 | *N* | 110 | 10 | 0 |  | 80 | 40 |  | 120 | 0 | 0 |  | 105 | 15 | 0 |  | 105 | 15 |
|  |  | *P* (%) | 91.67 | 8.33 | 0 |  | 66.67 | 33.33 |  | 100.00 | 0 | 0 |  | 87.50 | 12.50 | 0 |  | 87.50 | 12.50 |
| BJFT | 58 | *N* | 107 | 9 | 0 |  | 114 | 2 |  | 116 | 0 | 0 |  | 63 | 53 | 0 |  | 63 | 53 |
|  |  | *P* (%) | 92.24 | 7.76 | 0 |  | 98.28 | 1.72 |  | 100.00 | 0 | 0 |  | 54.31 | 45.69 | 0 |  | 54.31 | 45.69 |
| SDJNan | 108 | *N* | 191 | 25 | 0 |  | 196 | 20 |  | 214 | 2 | 0 |  | 166 | 50 | 0 |  | 166 | 50 |
|  |  | *P* (%) | 88.43 | 11.57 | 0 |  | 90.74 | 9.26 |  | 99.07 | 0.93 | 0 |  | 76.85 | 23.15 | 0 |  | 76.85 | 23.15 |
| SDJNing | 44 | *N* | 84 | 4 | 0 |  | 85 | 3 |  | 2 | 1 | 85 |  | 76 | 12 | 0 |  | 76 | 12 |
|  |  | *P* (%) | 95.45 | 4.55 | 0 |  | 96.59 | 3.41 |  | 2.27 | 1.14 | 96.59 |  | 86.36 | 13.64 | 0 |  | 86.36 | 13.64 |
| HNSS | 36 | *N* | 33 | 29 | 10 |  | 46 | 26 |  | 60 | 2 | 10 |  | 36 | 26 | 10 |  | 31 | 41 |
|  |  | *P* (%) | 45.83 | 40.28 | 13.89 |  | 63.89 | 36.11 |  | 83.33 | 2.78 | 13.89 |  | 50.00 | 36.11 | 13.89 |  | 43.06 | 56.94 |
| HNSY | 91 | *N* | 143 | 39 | 0 |  | 148 | 34 |  | 182 | 0 | 0 |  | 160 | 22 | 0 |  | 156 | 26 |
|  |  | *P* (%) | 78.57 | 21.43 | 0 |  | 81.32 | 18.68 |  | 100.00 | 0 | 0 |  | 87.91 | 12.09 | 0 |  | 85.71 | 14.29 |
| GDGZ | 42 | *N* | 24 | 60 | 0 |  | 71 | 13 |  | 84 | 0 | 0 |  | 26 | 58 | 0 |  | 26 | 58 |
|  |  | *P* (%) | 28.57 | 71.43 | 0 |  | 84.52 | 15.48 |  | 100.00 | 0 | 0 |  | 30.95 | 69.05 | 0 |  | 30.95 | 69.05 |
| GXNN | 53 | *N* | 64 | 42 | 0 |  | 60 | 46 |  | 106 | 0 | 0 |  | 63 | 43 | 0 |  | 63 | 43 |
|  |  | *P* (%) | 60.38 | 39.62 | 0 |  | 56.60 | 43.40 |  | 100.00 | 0 | 0 |  | 59.43 | 40.57 | 0 |  | 59.43 | 40.57 |
| Total | 1548 | *N* | 2452 | 634 | 10 |  | 2822 | 274 |  | 2979 | 11 | 106 |  | 1518 | 1550 | 28 |  | 1501 | 1595 |
|  |  | *P* (%) | 79.20 | 20.48 | 0.32 |  | 91.15 | 8.85 |  | 96.22 | 0.36 | 3.42 |  | 49.03 | 50.06 | 0.90 |  | 48.48 | 51.52 |

Abbreviations: *N*, number of alleles; *P*, frequency of the allele.
